# Supplementary material for: Patterns of Intron Gain and Loss in Fungi
Source: PLoS Biol. 2004 Nov 30;2(12):e422. doi: 10.1371/journal.pbio.0020422 (PMC532390; doi:10.1371/journal.pbio.0020422)
Supplement: Table S1 — Also available at http://genes.mit.edu/NielsenEtAl/. (4.3 MB ZIP). [file pbio.0020422.st001.zip › NielsenEtAl/html/217.html]

AN0183.1.NCU08824.1.MG06153.1.FG09776.1


```
 CLUSTAL W (1.82) Multiple Sequence Alignments - Introns Inserted


Sequence 1: MG06153.1	294 aa
Sequence 2: FG09776.1	320 aa
Sequence 3: NCU08824.1	295 aa
Sequence 4: AN0183.1	309 aa
Alignment Length: 340 aa
Number Identitical Residues: 125 aa
Alignment Score (without introns) 6346


MG06153.1 	MA--SADERN-SRTVHT~AACLIIGD------EVLGGK0TVD0TNSNTVAKWCFELGI~N
NCU08824.1	MYKTPPSSRLPSQGRKS~APHGITAKSTAQLLRYLPRS0TKD0TNSNYMAKWCFNLGI0N
FG09776.1 	MAPVSADERN-SRTIST~AACLIIGD------EVLGGK0TVD0TNSAYLAKWCFNLGI0S
AN0183.1  	----------MKYSVGR2AGQKLIGQ------------~TVD~TNSAFFARYCFSLGI~Q
          	           .      *   : ..             * * ***  .*::**.*** .

MG06153.1 	LKRIEVIEDDESEIIEAVRRMSDRYDFVVTS2GGIGPT2----HDDITY-----Q~SIAK
NCU08824.1	LKRIEVIADDEDEIVEAVRRMSDRYDFVVTS2GGIGPT~----HDDITY-----Q~SIAK
FG09776.1 	LKRIEVIADDESEIIEAVRRMSDGYDFVVTR~QVFKRT~LVYQAANINQWWNWTY0SIAT
AN0183.1  	LKRIEVIADEEEEIIEAVRRMSKNYDFVVTS~GGIGPT2----HDDITY-----S~SIAR
          	******* *:*.**:*******. ******    :  *       :*.        *** 

MG06153.1 	AFGLDLKLHNDAYERMKKLSRPHPSQPKFNWDEDSPAKRAKLRMVELPTDESRDMDKQFL
NCU08824.1	AFGLPLKLNEEAFKLMKKLSVPRKGEPPFNWDEPSPVLTAKLRMVELPTDVNRDLAKQFL
FG09776.1 	AFNLPLKLHQETFEKMKLLSKPHPNQPTFDWEVDSPARRAKLRMAELPTDESRDLKKQVL
AN0183.1  	AFNLPLKLHEAAFERMKKLTKPHALTPDFSWDTPSPALTARLRMVELPFDASLPAESQAI
          	**.* ***:: ::: ** *: *:   * *.*:  **.  *:***.*** * .    .* :

MG06153.1 	FPREELWVPVCVVNGNVHILPGVPKLF1VSLLEGLKPYIVPRLVDPEGKGICRVMISTPL
NCU08824.1	FPCKELWVPVAVVNGNVHILPGVPRLF1EKLLDGLKPSIQPRLVDPDGKGITRVTISTPL
FG09776.1 	FPQEDLWVPVSVVNGNIHILPGVPRLF1QRLLEGLKSHVLPRLSDPEGKGTHRVLFSTPV
AN0183.1  	FVSDDMWVPIAIVNGNVHILPGVPRLF~ERLLEHLRPSLLPRLVNPEGKGIYRYLFSTPL
          	*  .::***:.:****:*******:**   **: *:. : *** :*:***  *  :***:

MG06153.1 	AESAMAAYLTELAAKVEPQGVKVGSYPRWNKKRNTVTLVGK2NKAFIESLIPEVTKNVQG
NCU08824.1	PESAIAAYLTELAARVEPKGVKVGSYPRWGEKHNTVTLVGK2DKDFLNSIAPEVSHYVKG
FG09776.1 	PESGVAEFLTTLAAKVEPKGVKVGSYPRWGKENNTVTLVGR2DLDYLESLVEEVEAGIQG
AN0183.1  	PESAVAPYLTDLATRTAARGVKVGSYPRWQKKRNTVTLVGT~DKEFLDSLVAEVEENVQG
          	.**.:* :** **::. .:********** ::.*******  :  :::*:  **   ::*

MG06153.1 	RVITVEGE-DDDPK--~--------DKD--EPVSQ---------------
NCU08824.1	QIVTTESDDDTSS---~---------------------------------
FG09776.1 	MRVTAESDTEEDPKQI~KKQATEDADKDTAKQVAQKP-------------
AN0183.1  	KKVSAEDELDSDTEEK2SLTMRYIFPPHLIIWDAIGLAVVILDLEGFSDI
          	  ::.*.: : ....  .               :   :    . .. :.
```
